# Regional changes in reactive hyperemic blood flow during exercise training: time-course adaptations

**DOI:** 10.1186/1476-5918-6-1

**Published:** 2007-01-12

**Authors:** Mahmoud A Alomari, Michael A Welsch

**Affiliations:** 1 Department of Allied Medical Sciences, Jordan University of Science & Technology, Irbid, Jordan; 2 Department of Kinesiology, Louisiana State University, Baton Rouge, Louisiana, USA

## Abstract

**Background:**

Few studies have examined the time-course of localized exercise training on regional blood flow in humans. The study examined the influence of handgrip exercise training on forearm reactive hyperemic blood flow and vascular resistance in apparently healthy men.

**Methods:**

Forearm blood flow and vascular resistance were evaluated, in 17 individuals [Age: 22.6 ± 3.5], in both arms, at rest and following 5 minutes of arterial occlusion, using strain gauge plethysmography, prior to training (V1) and every week thereafter (V2-5) for 4 weeks. Handgrip exercise was performed in the non-dominant arm 5 d/wk for 20 minutes at 60% of maximum voluntary contraction, while the dominant arm served as control.

**Results:**

Resting HR, BP, and forearm blood flow and vascular resistance were not altered with training. The trained arm handgrip strength and circumference increased by 14.5% (p = 0.014) and 1.56% (p = 0.03), respectively. ANOVA tests revealed an arms by visit interaction for the trained arm for reactive hyperemic blood flow (p = 0.02) and vascular resistance (p = 0.009). Post-hoc comparison demonstrated increased reactive hyperemic blood flow (p = 0.0013), and decreased post-occlusion vascular resistance (p = 0.05), following the 1^st ^week of training, with no significant changes in subsequent visits.

**Conclusion:**

The results indicate unilateral improvements in forearm reactive hyperemic blood flow and vascular resistance following 1 week of handgrip exercise training and leveled off for the rest of the study.

## Background

Structural and functional changes in the arterial system following exercise training are well documented. These adaptations are associated with reduced vascular resistance, improved blood delivery and diffusion in the contracting muscles. Additionally, they contribute to improved vascular modulation and exercise performance during subsequent exercise sessions. The mechanism(s) involved in these adaptations are not fully understood, but include alterations in several vascular controllers including those specific to the arterial endothelium and/or smooth muscle [1-4].

Recent studies report training-induced adaptations are observed following 1 week of exercise training in animal models [5,6], and in human single large conduit arteries (i.e. brachial) [7]. Interestingly, the rapid training adaptations have been reported in very specific sections of the vasculature [7]. We are unaware of any published studies in humans that have examined the time-course of regional, rather than site-specific, vascular adaptations to exercise training, which certainly involve distinct vasoregulatory mechanisms [2]. Ultimately, such information may increase our understanding of the manner in which the vasculature adapts and may contribute to the development of appropriate strategies to protect vascular health. Therefore the purpose of this study was to examine the time-course of forearm vascular adaptations to handgrip exercise training.

It is hypothesized that 4 weeks of handgrip exercise training will result in an increase in reactive hyperemic blood flow, subsequent to reduction in vascular resistance, in the trained arm only. Additionally, given the recent findings in animal and single human large conduit arteries, it is anticipated that regional vascular adaptations will occur early in the training program.

## Methods

### Participants

Apparently healthy sedentary young adult males were recruited to participate in the study. Individuals with manifestations of cardiovascular, metabolic, orthopedic or neurological disease or on any medication affect the results of this study were excluded. The study was completed in 4 weeks, and involved once a week evaluation session and 5-time-a-week handgrip exercise training. Prior to and at the end of each week, participants underwent vascular function evaluation in the dominant and non-dominant forearms. The primary outcomes of this study were: (1) Reactive hyperemic blood flow, defined as blood flow vascular response to upper arm arterial occlusion and; (2) forearm post-occlusion vascular resistance, defined as mean arterial blood pressure divided by reactive hyperemic blood flow [4]. Additionally, handgrip strength, and hemodynamic measures (i.e. blood pressure, heart rate and heart rate variability) were assessed. Following the completion of the initial assessment, each subject was asked to report to the exercise physiology laboratory 5 days/week to perform 20 minutes of handgrip exercise at a cadence of 1 contraction every 4 seconds at 60% of maximum handgrip strength. The non-dominant arm was trained while the dominant arm served as control.

### Experimental Protocol

Blood flow measures were obtained in the dominant and non-dominant forearms using mercury strain gauge plethysmography (model EC5R, D. E. Hokanson Inc, Bellevue, WA, 99) at rest and following occlusion [8], and at the beginning of the study, at the end of each week (but prior to that day's exercise session), throughout the study period. The technique is noninvasive, and based on the assumption that alterations of pressures in strategically placed cuffs allows examination of the rate of change in limb volume thought to reflect vascular function indices including blood flow and vascular resistance [9,10].

Prior to the experiment, blood pressure cuffs were positioned around the participant's upper arm and wrist, and a mercury-in-Silastic strain gauge placed around the forearm approximately 10 cm distal to the olecranon process [8]. Resting forearm blood flow was obtained following 15 minutes of supine rest. Immediately before the measurements, hand circulation was occluded for 1 minute by inflating the cuff at the wrist to 240 mmHg. Forearm blood flow was obtained using an upper arm venous occluding pressure of 7 mmHg below diastolic blood pressure [8]. Subsequently, reactive hyperemic forearm blood flow was examined following arterial occlusion achieved by inflating the upper arm cuff to 240 mmHg for 5 minutes. Forearm blood flow was then determined as described above. The same measures were then performed in the other arm. Prior to, during and following each procedure blood pressure and heart rate were obtained.

Short-term measures of heart rate variability were obtained in the last 5 minutes of supine rest, and prior to the vascular assessments. The ECG electrodes were placed on the chest and interfaced with a Biopac MP100 and its companion software Acqknowledge (model MP100A, Biopac Inc., Santa Barbara, CA) to allow for continuous data acquisition. Five minutes of ECG data were analyzed at rest for mean heart period (mean R-R interval), standard deviation (SDNN), low frequency power (LF) and high frequency power (HF) using the guidelines set forth by the Task Force of the European Society of Cardiology and the North American Society of Pacing and Electrophysiology [11]. To standardize physiological studies, the Task Force recommends that the use of short-term recordings of 5 minutes under physiologically stable conditions may be an appropriate reflection of sympathovagal balance when frequency domains are processed [11]. The heart rate variability data were obtained at the beginning and at the end of the study period.

The participant's forearm circumferences and handgrip maximal voluntary contraction were evaluated at baseline and at the end of each week throughout the study period. Forearm circumference was examined using a weighted measuring tape 10 cm distal to the midpoint between the lateral epicondyle and olecranon process. Handgrip strength was evaluated using a handgrip dynamometer (Lafayette instruments, OH, 02) with the participant upright, but slightly bent forward at the waist. The test involved an all-out gripping effort for 3 seconds, without movement of the arm. The average of three consecutive trials was used as the measure of strength.

### Data Analyses

Resting blood flow was recorded at a paper speed of 5 mm/seconds and values were derived from the slope drawn at a best-fit tangent using the first 2–3 pulses. Calculations were made as a function of 60 seconds divided by the horizontal distance (mm) needed for the slope to rise vertically from baseline to the top of the recording paper and multiplied by the full chart range [9,10]. Forearm reactive hyperemic blood flow was recorded at a paper speed of 25 mm/second. Analyses were performed using a slope drawn at a best-fit tangent to the curves of the first 2-pulse flows post-cuff release. Blood flows were then calculated from 60 seconds multiplied by the paper speed, and divided by the horizontal distance (mm) needed for the volume slope to increase by 20 mm vertically [8]. Forearm vascular resistance was then determined by dividing mean arterial pressure by the blood flow. Vascular indices were expressed in ml·100 ml tissue^-1^·min^-1^.

### Statistical Analyses

All statistical analyses were completed using SAS (version 9.00, Cary, NC) and SPSS (version 11.00, Chicago, Ill) statistical programs. In order to examine the influence of 4-weeks of handgrip exercise training on the trained arm reactive hyperemic responses, a 2 (Arms) * 5 (visit 1-visit 5) split-plot ANOVA was used. A paired t-test was used to compare between pre and post training measures of blood pressure, heart rate, heart rate variability indices, arm circumference, and handgrip strength. Alpha was set a-priori at *p*<0.05.

## Results

### Participant characteristics

Twenty-two men participated in the study. Five participants did not complete the study due to personal decision (3 individuals) or lack of compliance (2 individuals). Participant descriptions are presented in Table 1.

**Table 1:** Subject characteristics

**Variable**	**Visit 1 (Pre Training)**
Age (yrs)	22.6 ± 14.4
Height (cm)	175.4 ± 6.6
Weight (kg)	75.5 ± 16.5
Control arm length (cm)	27.5 ± 0.4
Trained arm length (cm)	27.4 ± 0.4

Values are mean ± SD.

### Hemodynamic, anthropometric and exercise measures

Hemodynamic, anthropometric and exercise measures before training (V1 Pre Training) and the end of the study (V5 End Training) are presented in Table 2. No significant changes in heart rate, blood pressure, and heart rate variability indices were observed. Moreover, there were no changes in anthropometric measures in the non-trained arm. In contrast, forearm circumference increased by 1.56% (p = 0.03) in the trained arm. Handgrip strength was not modified in the non-trained arm, whereas the trained arm showed a 14.5% (p = 0.014) increase in maximal voluntary contraction.

**Table 2:** Hemodynamics, Heart Rate Variability, Strength and Circumference before and after 4-week handgrip exercise training

**Variable**	**Visit 1 (Pre Training)**	**Visit 5 (End Training)**
HR (bpm)	58.2 ± 6.6	57.5 ± 3.8
SBP (mmHg)	112.6 ± 9.9	111.1 ± 8.7
DBP (mmHg)	60.7 ± 7.8	59.7 ± 7.4
MAP (mmHg)	78.0 ± 33.4	76.8 ± 28.9
SDNN (ms)	73.4 ± 16.9	79.6 ± 16.5
LF power	36.9 ± 5.4	37.2 ± 5.8
HF power	63.1 ± 5.4	62.8 ± 5.8
Control handgrip strength (kg)	40.6 ± 12.0	42.8 ± 3.2
Trained handgrip strength (kg)	36.1 ± 8.7	41.3 ± 1.7*
Control arm circumference (cm)	26.3 ± 2.1	26.5 ± 2.1
Trained arm circumference (cm)	25.6 ± 2.5	26.0 ± 2.1*

Values are mean ± SD. * = p < 0.05 vs. visit 1

### Effect of training on vascular function

**Figure 1:**
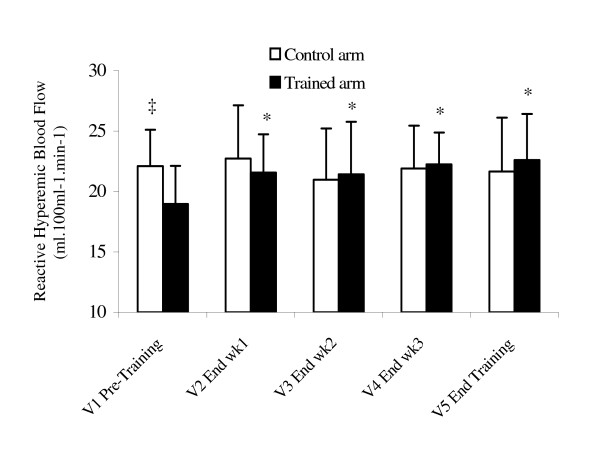
Changes in forearm reactive hyperemic blood flow. Values are mean ± SD. * = p < 0.05 vs. V1 Pre Training; ‡ = p < 0.05 vs. Trained arm.

**Figure 2:**
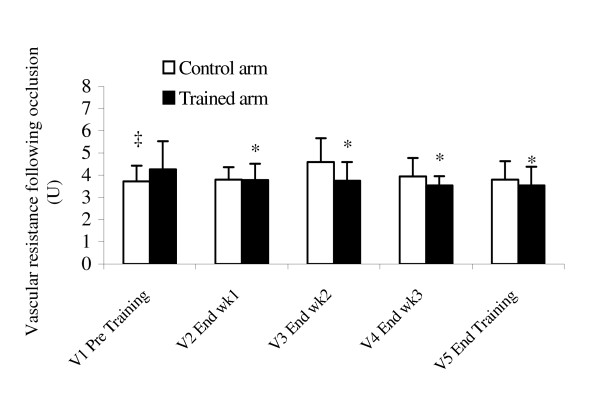
Changes in forearm reactive hyperemic vascular resistance. Values are mean ± SD. * = p < 0.05 vs. V1 Pre Training; ‡ = p < 0.05 vs. Trained arm.

Table 3 demonstrates that 4-weeks of handgrip exercise training did not change resting blood flow or vascular resistance. However, as depicted in Figures 1 and 2, there was a significant increase in reactive hyperemic blood flow and decrease in post occlusion vascular resistance following training. In fact, the 2 * 5 split-plot ANOVA test revealed a significant interaction between arms and visits for reactive hyperemic blood flow (p = 0.02) and post occlusion vascular resistance (p = 0.009) in the trained arm only. Subsequent post-hoc comparisons demonstrate increased reactive hyperemic blood flow (p = 0.0013), and decreased forearm vascular resistance (p = 0.05) following the 1^st ^week (V2), which is maintained throughout the rest of the visits.

**Table 3:** Resting blood flow and vascular resistance

	**Control arm**		**Trained arm**	
**Variable**	**Visit 1 (Pre Training)**	**Visit 5 (End Training)**	**Visit 1 (Pre Training)**	**Visit 5 (End Training)**

**Resting Blood flow **(ml · 100 ml tissue^-1 ^· min^-1^)	2.9 ± 0.8	2.6 ± 0.8	3.0 ± 0.8	3.0 ± 0.8
**Vascular resistance at rest **(U)	29.4 ± 9.5	31.7 ± 9.5	28.1 ± 9.5	28.6 ± 9.5

Values are mean ± SD. * = p < 0.05 vs. visit 1; ‡ = p < 0.05 vs. Trained arm.

## Discussion

The main finding of this study was a unilateral improvement in reactive hyperemic forearm blood flow following handgrip exercise training. Uniquely, regional vascular improvements were evident after the 1^st ^week of training and appeared to plateau during the remainder of the training period, confirming data from animal models [5,6] as well as from human studies involving single large conduit artery reactivity [7].

Four weeks of handgrip training resulted in a 19% increase in regional reactive hyperemic blood flow after 5 minutes of occlusion. This increase confirms previous data from our laboratory (21%) [1] as well as others (25%) [4] using a similar frequency, intensity, and duration of training. The increase in reactive hyperemic blood flow in the trained arm occurred without alterations in the contra-lateral arm. This finding coupled with evidence that cardiovascular hemodynamics (i.e. blood pressure and heart rate), and measures of autonomic balance (SDNN, LF, and HF) did not change suggests that these adaptations are locally modulated. Although it is beyond the scope of this study, it is hypothesized that the muscle contraction and relaxation phases associated with handgrip exercise serves as the stimulus for the adaptive processes in the trained region. These adaptations are aimed at increasing vascular conductance and oxygen and nutrient delivery to working skeletal muscle during subsequent exercise bouts [12]. The manner in which the repetitive mechanical muscle contractions affect regional vascular control mechanisms is not entirely understood. However, the 4-week training program may have resulted in regional adaptations including endothelial, metabolic [2], neural [13], and structural [14] modifications, while the contribution of muscle mass and type [15] and the venous system [16] cannot be excluded.

As depicted in Figure 1, reactive hyperemic blood flow increased 13% after the 1^st ^week of training, followed by a more gradual increase over the remainder of the training period. These results confirm data of a single large conduit artery reported previously by our laboratory [7]. The mechanism(s) contributing to the rapid adaptations in regional blood flow are not clear, but will be discussed briefly.

Changes in muscle mass probably did not account for the rapid increase in reactive hyperemic flow responses. The 4-week training program did result in a modest increase in forearm circumference (1.56%) as well as 14.5% in handgrip strength, but the small changes in forearm circumference were observed at the end of the study not after one week. It is possible the improvements are the consequence of modifications in vascular smooth muscle responses to local metabolites released during the occlusion period. In fact, Delp & Laughlin suggest improved blood flow may be secondary to an increase in smooth muscle sensitivity attributed to changes in metabolic vasoregulators [17]. However, such changes are usually not evident until 4 weeks after training [17].

Several investigators have suggested a role of the vascular endothelium (in particular the nitric oxide pathway) in the rapid adaptations of the vasculature following initiation of exercise training. It would appear the results of this study are consistent with the hypothesis advanced by McAllister and Laughlin who speculate that vascular endothelial function is enhanced after just a few days of training and that such adaptations could serve to buffer the increase in shear stress experienced during exercise [12]. We support this hypothesis but also recognize the limitation of the current study as reactive hyperemia may not be influenced to a large degree by nitric oxide [18]. Instead, the magnitude of reactive hyperemia is probably more affected by prostaglandins and adenosine [19]. Interestingly, data are emerging to suggest that an acute bout of exercise has a significant effect on the release of vasodilator substances such as prostaglandins, adenosine and nitric oxide [20]. It is speculated these molecules may represent the signal for increased capillary formation to accommodate greater exercise capacity [20]. In fact, even more recent data indicate that acute exercise stimulates vascular endothelial growth factor, which is coupled to muscle capillary supply [21].

Thus the rapid improvements in the reactive hyperemic response and vascular resistance in this study may be a consequence of changing shear rates during the muscle contraction and relaxation phase associated with handgrip exercise. As a result of the changing shear rate, vasodilators (such as prostaglandins, adenosine and nitric oxide) are released following each contraction and stimulate vascular endothelial growth factors, which are coupled to muscle capillary supply. In fact, Suzuki et al. report an increased capillary cross-sectional area after only 1 week of exercise training, and attributed this to recruitment of "pre-existing" capillaries [22]. In addition, Waters et al. attributed the increased capillary density after only 7 days of training to angiogenesis [23]. The observation that angiogenesis-related gene expression rapidly attenuates with training [24] may explain the plateau in the reactive hyperemic responses over the remainder of the training period. Future studies need to determine if an increase in volume of training (intensity, duration, frequency) would further stimulate vascular adaptations.

Finally, recent evidence suggests the improvements may be mediated through the venous system. Perhaps the exercise training resulted in increased forearm venous emptying, secondary to regional elevation in sympathetic drive, or improved skeletal muscle pump [25] which has been shown to contribute to a greater arterial flow response [16]. A greater venous emptying may also facilitate driving blood towards the delivery segments of the circulation [25,26].

No one mechanism has been singled out as the primary explanation for improved blood flow after training. Moreover, the contributions of each proposed mechanism to the increase in blood flow after training is currently also not fully known. However, the current advancements in vascular physiology indicate that these abovementioned mechanisms collectively enhance vascular function in the trained body. Thus more studies are certainly needed to further improve our understanding of vascular adaptations to exercise training.

The unilateral improvements in vasoreactivity, once again, confirm the concept of specificity of training. Consequently, these findings support the need for an exercise training program targeting the heart and major muscle groups as advocated by ACSM [27].

## Conclusion

Data from the current study demonstrate a unilateral improvement in post occlusion reactive hyperemic blood flow after the 1^st ^week of handgrip exercise training and appeared to plateau over the remainder of the study. The unilateral responses to handgrip exercise training further advocate the importance of whole-body exercise training programs targeting major muscle groups as recommended by recent ACSM exercise guidelines. Finally, the rapid adaptations demonstrated herein are intriguing and suggest that exercise training might be an effective counter-measure to protect the vasculature from the constant barrage of daily stresses, such as high fat meals, cigarette smoke and other stresses.

## Competing interests

The author(s) declare that they have no competing interests.

## Authors' contributions

MA: coordinated and executed study design and contributed to the manuscript

MAW: conceived of study design and contributed to the manuscript

All authors read and approved of the final manuscript
